# The Impact of Economic Growth and Air Pollution on Public Health in 31 Chinese Cities

**DOI:** 10.3390/ijerph16030393

**Published:** 2019-01-30

**Authors:** Ying Li, Yung-ho Chiu, Tai-Yu Lin

**Affiliations:** 1 Business School, Sichuan University, Wangjiang Road No. 29, Chengdu 610064, China; liyinggs@scu.edu.cn; 2 Department of Economics, Soochow University, 56, Kueiyang St., Sec. 1, Taipei 10048, Taiwan; eickyla@gmail.com

**Keywords:** environmental efficiency, Epsilon-Based Measure, health expenditure, network SBM

## Abstract

The rapid economic growth of China in the last twenty years has caused a commensurate rise in atmospheric pollution which has had an impact on both the environment and public health. Since 2013, SO_2_, CO_2_ and nitrogen oxide levels have reached a level that may cause climate change and have adverse effects on the health of the local residents. Past environmental efficiency analyses have rarely examined economic development, air pollution and health as interacting systems; therefore, this study used a new two-stage DEA model, the Modified Undesirable EBM Two Stage DEA (Epsilon-Based Measure) to explore the environmental, economic and health efficiencies in thirty-one major cities in China. The results were as follows: while all cities needed to improve their GDP, the environmental efficiencies were continuing to rise in most cities. The health efficiency index indicated that disease efficiency had increased in most cities but declined in one third; therefore, it is necessary to strengthen treatment. The respiratory disease treatment efficiency in most cities was rising, and the room for improvement had significantly reduced. There were improvements in the mortality rate in 15 cities; however, the mortality rate treatment efficiency declined in 11 cities.

## 1. Introduction

To accompany its growing economy over the past few decades, China has consumed large amounts of coal and oil, which has resulted in a significant drop in air quality in many cities and has had a significant impact on the environment and public health. The World Meteorological Organization (WMO) reported that the current CO_2_ concentrations in the Earth’s atmosphere were now 145% of the level before industrialization [1]. This rapid rise in CO_2_ has resulted in climate change effects such as the rapid loss in sea ice through melting, which has begun to endanger coastal cities and affect river flows, which in turn have affected production activities.

Many countries are now seeking to limit their carbon emissions, and China is also aiming to have “ambient air quality standards” [2] by 2040 for half the population. Because air pollution is already a major threat to public health and the impact of SO_2_, NO_2_, PM_2.5_ and CO_2_ on respiratory and cardiovascular diseases especially in children can be quite severe.

Environmental pollution research is generally linked to the assessment of the economy and energy efficiency. Zhou et al. [3] used non-oriented DEA to examine the carbon emission environmental performance in eight regions—OECD member countries, the Middle East, countries of the former Soviet Union, the non-OECD European countries, Asian countries outside China, China, Latin America, and Africa—by evaluating the non-increasing return to scale and variable return to scale efficiencies in each region. Sueyoshi and Mika [4] used the original non-radial DEA model to study the effect of the US Clean Air Act (CAA) on acid-induced gases (NO_x_) and found that under the CAA regulations, acid rain was effective in controlling the SO_2_ and NO_x_ emissions from U.S. coal-fired power plants. Shi et al. [5] used a Radial DEA model to analyze China’s energy efficiency, finding that the energy efficiency in eastern China was the best. Choi et al. [6] adopted a slack-based DEA to analyze China’s energy efficiency and found that China’s carbon dioxide efficiency was poor. Zhang and Choi [7] used an SBM-DEA to study the environmental efficiencies in various provinces in China and found that most provinces had low energy efficiencies. Sueyoshi and Mika [8] used DEA and the Malmquist index to examine the frontier changes between multiple periods, and studied the relationship between fuel mixtures, electricity, and carbon dioxide in 10 industrial countries, finding that the nuclear power generation in France, the water resources in The Netherlands, and renewable energy were important for the sustainable development of the society. Wu et al. [9] used the Radial DEA and Malmquist methods to explore the energy efficiency of China’s regions and found that the average energy efficiency in eastern and central China was higher. Chang [10] used Radial DEA to explore EU energy efficiency and found that the increase in energy intensity was related to a decrease in the energy intensity need for improvements. Wang and Wei [11] used a DDF model to analyze China’s energy efficiency and found that the energy efficiency was affected by the significant growth in carbon dioxide emissions. Pang et al. [12] used SBM DEA to analyze efficiencies in 87 countries and found that European countries were more efficient in emissions reduction and energy efficiency. Guo et al. [13] employed dynamic DEA models to assess the intertemporal efficiencies in OECD countries, and found that most countries had improved efficiencies. Georgiev et al. [14] evaluated the Environmental Kuznets Curve (EKC) for six air pollutants, namely SO_x_, NO_x_, CO, VOC CO_2_, and GHG, in OECD countries. Some air pollutant emissions did not follow the shape of EKC, but SO_X_ emissions show a U-shaped Environmental Kuznets Curve. He et al. [15] presented an assessment of the marginal abatement cost of the industrial sector in China. Li et al. [16] measured the damage caused by PM_10_ and SO_2_ in 74 cities in China in 2015. The above two air pollutants could cause economic losses related to health issues equal to 1.63 and 2.32% of the GDP. Wang et al. [17] estimated the relation between CO_2_ emissions and economic growth. The results showed that urbanisation level has a more significant effect on carbon emission than Gross Domestic Product per capita and energy structure. Xie et al. [18] used a computable general equilibrium (CGE) model and the latest non-linear exposure−response functions to assess the impact energy on China’s national and provincial economies, and found that in provinces with high concentrations of energy, the effect on health has had a significant impact on the economy. It was estimated that China’s GDP losses would be 2% by 2030, and the medical expenses would be around US $25.2 billion.

Environmental pollution and health have had two main research directions_ an assessment of the impact of environmental air pollutant exposure on health, and the impact of environmental air pollution on the health of children and the elderly. Liu et al. [19] investigated the effect of socioeconomic variables (education, income, life satisfaction, etc.) and air pollution on health in different regions (East, Central, West) of China in 2015. The results show that good air quality in spring and summer has a positive effect on health, but the bad air quality in autumn and winter has a negative effect on health. Zanoni et al. [20] investigated the effect of civilian activities on Indoor Air Quality (IAQ) in Italy in 2013. The results show that church services, public transportation, and A/C in cars do have an effect on IAQ. Kelly and Fussell [21] concluded that particulate matter (PM) has not only had a major impact on health over the past 10 years, but was also associated with many diseases, with the PM_2.5_ concentrations having a particularly significant impact on health. Fischer et al. [22] explored the relationship between long-term exposure to air pollution, urban living, and societal health using the Land Use Regression (LUR) method and found that: (1) For every 10 μg/m^3^ increase, PM_10_ and NO_2_ were significantly associated with non-accidental mortality; (2) PM_10_ was related to circulatory system mortality, but NO_2_ was not; and (3) Long-term exposure to PM_10_ and NO_2_ was associated with (non-accidental causes) mortality in The Netherlands for people over 30. Khafaie et al. [23] focused on the links between air pollution and health and reviewed the different estimation methods, finding that the quality of the exposure measurements was a key determinant in epidemiological studies and that available exposure data collection methods were often related to the design determinants. Khafaie et al. also conducted a historical review of air pollution research [24], providing a comprehensive analysis of the health effects of harsh air quality, evaluating the rational biological mechanisms of the impact of air pollution, and identifying specific air pollutants.

Tainio et al. [25] analyzed the relationship between different levels of exercise and air pollution related mortality, and found that in an area that has a PM_2.5_ concentration of 100 μg/m^3^, the benefit from riding for one and a half hours a day or walking for more than 10 h a day was less than the damage from the air pollution, and in areas with a lower PM_2.5_ concentration, the benefit of cycling for 3 h and a half rather than staying home was greater than the damage from the air pollution. Jose et al. [26] measured the effect of climate on citizen’s health in two areas (Kensington and Chelsea) in London. The results showed that MICROSYS-CFD model can be benefit for urban atmosphere assessment and environmental policy design. Torretta et al. [27] discussed the pollution caused by particulate matter in Italy and provide policy suggestion. Schiavon et al. [28] assessed the effect of NO_x_ on health in Italy and provide governance recommendation to avoid health risk. Schiavon et al. [29] simulated the NO_x_ emissions of road traffic. The results show that high emission concentrations can affect the human body.

Cohen et al. [30] explored the spatial and temporal trends in mortality and diseases caused by air pollution at global, regional, and national levels. Through satellite estimation, a chemical transmission model, and ground level measurements, it was found that environmental PM_2.5_ ranked fifth among all mortality risk factors, with exposure to PM_2.5_ causing 42 million deaths, which was 7.6% of the total global mortality, and 1031 million disability-adjusted life years (DALYs). Johansson [31] used transport models to select commuter preference data for Stockholm County to analyze the effect of NO_x_ and black carbon (BC) on the mortality rate, and showed that a 7% decrease in the average exposure to nitrogen oxides and black carbon (BC) would reduce the relative risk of mortality by 8%. Newell et al. [32] explored the health effects of exposure to air pollutants, and found that a 10 μg/m^3^ increase in PM_2.5_ increased the risk of cardiovascular mortality by 0.47% and respiratory mortality by 0.57% (0.28–0.86), and a 10 μg/m^3^ increase in PM_10_ increased the risk of cardiovascular mortality by 0.27% (0.11–0.44) and respiratory mortality by 0.56% (0.24–0.87). Kinney [33] reviewed research on the effects of climate change on air quality and human health and found that climate and weather had a large impact on the spatial and temporal distribution of air pollution concentrations; for example, at higher ambient temperatures, ozone and PM_2.5_ emissions increased, and ozone formation was faster in high sunlight and high temperatures. Lua et al. [34] measure the effect of the PM_2.5_ on health in China from 2001 to 2017. The PM_2.5_ is related to the mortality rate in urban. The reduction of the PM_2.5_ can brings the benefit about $193,800 in 2017.

Zigler [35] used causal inference methods and a spatially hierarchical regression model to investigate the impact of environmental fine particulate matter in 2005 (which did not meet the 1997 national environmental air quality standard) on environmental PM_2.5_, and found that at all research sites, any reductions in environmental PM_2.5_ and health insurance could not be attributed to the substandard conditions in eastern United States. A hierarchical analysis showed that if PM_2.5_ reduced to levels beyond the regional measures, mortality, chronic obstructive pulmonary disease, heart failure, ischemic heart disease, and respiratory infections would be significantly reduced.

Some research has discussed the impact of environmental air pollution on the health of children and the elderly. Frischer et al. [36] used regression analysis to estimate the links between air pollutants and lung function in 1,150 children in two Austrian counties from 1994–1996, and found that ozone had a significant negative effect on the first second of forced expiration (FEV_1_), forced vital capacity (FVC), and maximal expiratory flow at 50% of the vital flow capacity (MEF_50_). However, PM_10_, SO_2_, NO_2_ were not found to have a significant negative effect on lung function. Ye et al. [37] used generalized linear models (GLMs) to research the effects of exposure to higher daily maximum temperatures and concentrations of air pollutants in Tokyo during the summer months of July and August from 1980 to 1995 on cardiovascular and respiratory diseases in people over 65 years old, and found that NO_2_ had a significant effect on angina, cardiac insufficiency, myocardial infarction, and acute bronchitis, and PM_10_ had a significant effect on asthma, chronic bronchitis, and pneumonia.

Lee et al. [38] used a generalized additive model (GAM) time series analysis to explore the effect of multiple air pollutants; sulfur dioxide (SO_2_), nitrogen dioxide (NO_2_), ozone (O_3_), carbon monoxide (CO) and airborne particles less than or equal to 10 μg/m^3^ in aerodynamic diameter (PM_10_); on the health of children under 15 years old in Seoul from 1997 to 1999, and found that nitrogen dioxide and ozone were the main factors for childhood asthma. Gauderman et al. [39] estimated the relationship between air pollution and lung function using linear regression in children in the fourth, seventh, and tenth grades in southern California in 1993, and found that PM_2.5_, PM_2.5–10_, PM_10_ and NO_2_ had significantly negative effects on the first second of forced expiration (FEV), forced vital capacity (FVC), maximal mid-expiratory flow (MMEF), and improved forced expiratory flow at 75% of vital capacity (FEF_75_); however, ozone was not found to have a significant negative effect on lung function.

Chen et al. [40] used generalized linear models (GLMs) to explore the association between particulate matter (PM) and hospitalization for chronic obstructive pulmonary disease (COPD) and found that PM_2.5_, PM_2.5–10_ and PM_10_ all had significant effects.

Penard-Morand et al. [41] used a cross-sectional study of 6672 children from 9–11 years old at 108 randomly selected schools in France to research the impact of air pollution on asthma and allergies, and found that lifetime asthma and lifetime allergic rhinitis were positively related to an increased exposure to SO_2_, PM_10_ and O_3_; however, no consistent positive association was found for NO_2_; and that long-term exposure to background ambient air pollution increased the prevalence of respiratory and atopic problems in children. Barnet et al. [42] conducted a meta- analysis on children in two cities in New Zealand and five cities in Australia from 1998–2001 and found an association between outdoor pollution and hospital admissions, with the results showing that the outdoor pollutants PM_2.5_, PM_10_, SO_2_, NO_2_ and O_3_ had significant effects on pneumonia and acute bronchitis for children less than 1 and 1–4 years old, on respiratory problems in all three groups, and on asthma in 5–14 year old. SO_2_ has also been found to have an effect on pregnant women by shortening pregnancies, and causing premature births and low birth weights (Choe et al., [43]). A study of long-term exposure to PM_2.5_ and mortality in the elderly found (Wang and Shi [44]) that for every 1 μg/m^3^ increase in atmospheric PM_2.5_, the hazard ratio for mortality (HR) was 1.021 (95% CI: 1.019 to 1.022).

However, there have been few studies that have considered the interactions between economic growth, environmental pollution, and community health. Most environmental pollution and economic analysis research has employed radial (such as Cooper, Charanes and Rhodes Model or Banker, Chames and Cooper Model) or non-radial (Slack Based Model) Data Envelopment Analyses (DEA) and Distance Function Models (DDFC Directional). However, the radial DEA model ignores non-radial slacks and non-radial DEA models ignore characteristics of the same proportion. As traditional environmental pollution and economic research have tended to employ one-stage DEA as the main methodology, health issues have generally been ignored. Therefore, to resolve the issues associated with radial and non-radial bias, this research proposes a Modified Undesirable EBM Two stage model.

The main contribution of this paper is that the economic, environmental, and health efficiencies are jointly analyzed, which avoids under- or overestimation. Data from 31 Chinese cities from 2013–2016 were examined in two stages: a production stage and a health stage. In the production stage, labor, fixed assets, and energy consumption were the input indicators Gross Domestic Product (GDP) as output indicator, linkage and health stage variable are CO_2_ and Air Quality Index (AQI). In health stage the health expenditure is input indicator; the output indicators are birth rate, respiratory system and mortality rate.

The remainder of this paper is organized as follows: Section 2 presents the Materials and methods; Section 3 is the Results and Discussion; Section 4 is Conclusions.

## 2. Materials and Methods

Data envelopment analysis (DEA) uses linear programming techniques to assess the relative efficiency of decision making units (DMU) based on a Pareto optimal solution concept. Charnes et al. [45] developed the data envelopment analysis CCR DEA model based on the “frontier” concept by Farrell [46]). Banker et al. [47] then extended the assumption for the returns to scale and proposed the BCC model. As both CCR and BCC are Radial DEA models that ignore non-radial slacks when evaluating efficiency values, Tone proposed the Slacks-Based Measure (SBM) in 2001 [48] using the slack variable as a basis for measurement that considered the slacks between the inputs and outputs to provide an SBM efficiency that employed a non-radial estimation and single scalar method, with the radial DEA model being represented by the CCR and BCC and the non-radial DEA model being represented by the SBM. However, both models had shortcomings as the radial DEA model ignored the non-radial slacks when evaluating the efficiency value, and the non-radial DEA (such as SBM) failed to consider the radial characteristics when evaluating the efficiency value slacks, and therefore characteristics with the same radial proportion were ignored. Therefore, to solve these shortcomings, Tone and Tsutsui [49] proposed the Epsilon-Based Measure (EBM) DEA model, that included investment and output orientations and a non-orientation.

When evaluating efficiency, traditional DEA models convert the efficiency between the two variables through the input and output, with the conversion process being a “black box”. Fare et al. [50] then proposed Network Data Envelopment Analysis (Network DEA), which considered a production process composed of many sub-production technologies, which were regarded as sub-decision units (Sub-DMU), using traditional CCR and BCC models to determine the optimal solution. Zhu [51] described the value chain process as a “black box” and believed that it contained some sub-processes that constituted the value chain system. When estimating system efficiency, each sub-process also requires an efficiency evaluation. Chen and Zhu [52], Hwang and Kao [53], Kao and Hwang [54], and Kao [55] divided the entire business process into sub-processes that linked each stage with intermediate outputs. The efficiency of each stage was then calculated separately under different conditions, and which sub-process led to system efficiency losses were analyzed. Tone and Tsutsui [56] proposed a weighted slack-based measures network data envelopment analysis model (weighted slack-based measures) in which the links between the various departments of a decision-making unit were used as the basis for the Network DEA model analysis, and each department was seen as a Sub-DMU, after which the SBM mode was used to find the optimal solution.

Although the EBM DEA model overcame the shortcomings of the radial and the non-radial DEA models, it is still unable to solve the issues associated with two-stage undesirable output, and while Network DEA models can solve the multi-stage problems, they fail to solve the radial and non-radial problems. Therefore, this paper proposes a modified EBM Two-stage DEA model based on Tone and Tsutsui’s [49] Two-stage and modified EBM DEA model.

### 2.1. EBM DEA, Network SBM DEA and Modified Undesirable EBM Two Stage DEA Framework

The Non-oriented EBM DEA description in Tone and Tsutsui [49] is as follows:

There are n DMUs, where *DMU_j_* = (*DMU*_1_,*DMU*_2_,…,*DMU_k_*,…,*DMU_n_*), there are m types of input *X_j_* = (*X*_1*j*_,*X*_2*j*_,…,*X_mj_*), and there are *s* outputs, where *Y_j_* = (*Y*_1*j*_,*Y*_2*j*_,…,*Y_sj_*); the efficiency of the DMU unit is therefore [49]

(1) $$\theta^{*}=\underset{0,\eta ,\lambda ,s^{-},s^{+}}{\min}\frac{\theta -\varepsilon_{x}\sum_{i=1}^{m}\frac{w_{i}^{-}s_{i}^{-}}{x_{i0}}}{\eta +\varepsilon_{y}\sum_{i=1}^{s}\frac{w_{i}^{+}s_{i}^{+}}{y_{i0}}}$$

Subject to:

$$\theta X_{0}-X_{\lambda }-S^{-}=0,\;\eta Y_{0}-Y_{\lambda }+S^{+}=0,\;\lambda_{1}+\lambda_{2}+\ldots +\lambda_{n}=1\;\lambda \geq 0,S^{-}\geq 0,\;\;\;\;S^{+}\geq 0$$

where *Y*: DMU output, *X*: DMU input, $S^{-}$: slack variable, $S^{+}$: surplus variable, $W^{-}$: weight of input i, $\sum^{\text{​}}W_{i}^{-}=1\;\left(\forall_{i}W_{i}^{-}\geq 0\right)$, $W^{+}$: weight of output *S*, $\sum^{\text{​}}W_{i}^{+}=1\;\left(\forall_{i}W_{i}^{+}\geq 0\right)$, $\varepsilon_{x}$: set of radial θ and non-radial slacks, $\varepsilon_{y}$: set of radial η and non-radial slacks.

For DMU0, when $\theta^{*}=1$, EBM is considered the most non-oriented efficient. If the DMU is inefficient, the following adjustments are needed [49]:

(2) $$X_{0}^{*}=X\lambda^{*}=\theta^{*}X_{0}-S^{-*}\;\;Y_{0}^{*}=Y\lambda^{*}=\eta^{*}y_{0}+S^{+}$$

### 2.2. Non-Oriented Network SBM DEA

In 2009, Tone and Tsutsui proposed a weighted slack-based measures (DEM) model to measure the overall efficiency of decision-making units and the efficiency of each department and included an SBM mode non-radial measure when the input and output items could not be adjusted in equal proportions. The network DEA model based on the weighted SBM is introduced in the following: Considering both the input and output slacks, Equation (3) represents non-oriented efficiency [49]:

Minρ0∗=∑k=1Kwk[1−1mk[∑i=1mksi0k−Xi0k]]∑k=1Kwk[1+1rk[∑r=1mksi0k+Yr0k]]

(3) $$\begin{array}{l} \sum_{k=1}^{K}w^{k}=1,\forall k\\ w^{k}\geq 0,\forall k \end{array}$$

According to Equation (4), the definition for the non-oriented department efficiency is as follows [49]:

(4) ρk=1−1mk[∑i=1mksi0k−∗Xi0k]1+1rk[∑sr0k+∗Yr0k],k=1,2,.....,K

where $s_{i0}^{k{-}^{*}}$ and $s_{r0}^{k{+}^{*}}$ are the optimal input and output slacks, $\rho_{k}^{*}=1$ indicates that the *k*-th department of *DMU_o_* is non-oriented efficient, and indicates that *DMU_o_* has non-oriented overall efficiency.

### 2.3. The Empirical Model for this Study: Modified Undesirable EBM Two Stage DEA Model

By combining the modified EBM DEA with a Two-stage DEA and undesirable factors, this paper proposes a modified EBM Two stage DEA model to evaluate the energy efficiency of 31 cities in China, which avoids the efficiency and need for improvement being under or overestimated.

The modified undesirable EBM Two stage DEA is as follows:

The number of DMUs is *n*, the number of divisions is *k*, where *DMU_j_* = (*DMU*_1_,*DMU*_2_,…,*DMU_k_*,…,*DMU_n_*). there are *m* input types *X_j_* = (*X*_1*j*_,*X*_2*j*_,…,*X_mj_*), and there are *s* outputs where *Y_j_* = (*Y*_1*j*_,*Y*_2*j*_,…,*Y_sj_*), therefore, the efficiency of the DMU unit is:

(4) $$\theta^{*}=\underset{0\eta ,\lambda ,s^{-},s^{+}g,s^{-b}}{\min}\frac{\sum_{k=1}^{K}W^{k}\left[\theta_{k}-\varepsilon_{xk}\sum_{i=1}^{m_{k}}\frac{w_{i}^{-k}s_{i}^{-k}}{x_{i0}}\right]}{\sum_{k=1}^{K}W^{k}[\eta_{k}+\varepsilon_{yk}[\sum_{i=1}^{S_{1k}}\frac{w_{i}^{+S_{1k}}s_{i}^{+gk}}{y_{i0}}+\sum_{i=1}^{S_{2k}}\frac{w_{i}^{-S_{2k}}s_{i}^{-bk}}{y_{i0}}]]}$$

Subject to:

$$\theta_{k}X_{0}^{k}-X_{\lambda }^{k}-S^{-k}=0,\;\eta_{k}Y_{0}^{k}-Y_{\lambda }^{+gk}+S^{+gk}=0\;\eta_{k}Y_{0}^{k}-Y_{\lambda }^{-bk}+S^{-bk}=0\;Z^{\left(k,\;h\right)}=Z^{\left(k,\;h\right)}\lambda^{h}\;(\forall \left(k,\;h)\right)\;\lambda_{1}^{k}+\lambda_{2}^{k}+\ldots +\lambda_{n}^{k}=1$$

$$\lambda^{k}\geq 0,\;S^{-k}\geq 0,\;S^{+gk}\geq 0,\;S^{-bk}\geq 0,\;\theta_{k}\leq 1,\;\eta_{k}\geq 1$$

where *Y*: DMU output, *X*: DMU input, $S^{-k}$: slack variable, $S^{+gk}$: surplus variable, $S^{-bk}$: surplus variable, $W^{-k}$: weight of input I, $\sum^{\text{​}}W_{i}^{-}=1\;\left(\forall_{i}\;W_{i}^{-}\geq 0\right)$, $W^{+k}$: weight of output *S*,$\;\sum^{\text{​}}W_{i}^{+S_{1k}}+\sum^{\text{​}}W_{i}^{-S_{2k}}=1\;\left(\forall_{i}\;W_{i}^{+k}\geq 0\right),$ $\varepsilon_{xk}$: set of radial θ and non-radial slacks, $\varepsilon_{yk}$: set of radial η  and non-radial slacks, and (*k*, *h*): the link from Division *k* to Division *h*.

The efficiency score for Division *k* is given by:

(6) $$\rho_{k}=\frac{\theta_{k}-\varepsilon_{xk}\sum_{i=1}^{m_{k}}\frac{w_{i}^{-k}s_{i}^{-k}}{x_{i0}}}{\eta_{k}+\varepsilon_{yk}[\sum_{i=1}^{S_{1k}}\frac{w_{i}^{+S_{1k}}s_{i}^{+gk}}{y_{i0}}+\sum_{i=1}^{S_{2k}}\frac{w_{i}^{-S_{2k}}s_{i}^{-bk}}{y_{i0}}]}$$

when $\rho_{k}^{*}=1$, the *k* is the department, *DMU_o_* is non-oriented efficient and when $\rho_{0}^{*}=1$, *DMU_o_* has non-oriented overall efficiency. The DMU and division overall efficiency is assessed as the non-oriented unit the efficiency value and the measurement unit selected by the input, output, and the intermediaries are independent of each other.

If an inefficient decision-making unit needs to achieve an optimal efficiency goal, the following adjustments are needed:

$$X_{0}^{*k}=X^{k}\;\lambda^{*k}=\theta^{*k}X_{0}^{k}-S^{-*k}$$

$$Y_{0}^{*k}=Y^{k}\lambda^{*\left(+gk\right)}=\eta^{*k}Y_{0}^{k}+S^{+gk}$$

$$Y_{0}^{*k}=Y^{k}\lambda^{*-bk}\;=\eta^{*k}Y_{0}^{k}+S^{-bk}$$

### 2.4. Fixed Assets, Labor, Energy Consumption, GDP, Health Expenditure, Birth Rate, Respiratory Diseases, and Mortality Rate Efficiency

In this model, we assess the relative efficiency of decision making units (DMU) based on a DEA method. DMU with good efficiency score merely means that it has a “good rank” in comparison. However, it does not indicate that these municipalities have excellent air quality in the real world. Precisely, the air quality of these municipalities is “comparatively better”, thus, these municipalities perform better than other cities in the research sample in air pollutant reduction. In realistic, most cities in China have a large room for improvement in air quality. Since the “Air Pollution Prevention Action Plan” was ordained in China, every city has worked hard on air pollution issues. The cities with good rank in this paper have more chance to succeed in environment protection, due to their comparatively higher air pollution treatment efficiency.

In addition to finding the overall efficiency, the efficiency value for a single input or output can be found. Hu and Wang’s [57] total-factor energy efficiency index was adopted to overcome any possible bias in the traditional energy efficiency indicators. There are ten key features in this present study: Fixed assets, Labor, energy consumption, GDP, Health Expenditure, Birth Rate, Respiratory Diseases, Mortality Rate, CO_2_ and AQI.

In this study, “I” represents area and “t” represents time. The ten efficiency models are defined in the following expression:

(7) $$\mathrm{Fixed}\;\mathrm{assets}\;\mathrm{efficiency}=\;\frac{\mathrm{Target}\;\mathrm{Fixed}\;\mathrm{Assets}\;\mathrm{input}\;\left(i,\;t\right)}{\mathrm{Actual}\;\mathrm{Fixed}\;\mathrm{Assets}\;\mathrm{input}\;\left(i,\;t\right)}$$

(8) $$\mathrm{Labor}\;\mathrm{efficiency}=\;\frac{\mathrm{Target}\;\mathrm{Labor}\;\mathrm{input}\;\left(i,\;t\right)}{\mathrm{Actual}\;\mathrm{Labor}\;\mathrm{input}\;\left(i,\;t\right)}$$

(9) $$\mathrm{Energy}\;\mathrm{consumption}\;\mathrm{efficiency}=\;\frac{\mathrm{Target}\;\mathrm{Energy}\;\mathrm{input}\;\left(i,\;t\right)}{\mathrm{Actual}\;\mathrm{energy}\;\mathrm{input}\;\left(i,\;t\right)}$$

(10) $$\mathrm{GDP}\;\mathrm{efficiency}\;=\frac{\mathrm{Actual}\;\mathrm{GDP}\;\mathrm{desirable}\;\mathrm{output}\;\left(i,\;t\right)}{\mathrm{Target}\;\mathrm{GDP}\;\mathrm{desirable}\;\mathrm{output}\;\left(i,\;t\right)}$$

(11) $$\mathrm{Health}\;\mathrm{Expenditure}\;\mathrm{efficiency}=\;\frac{\mathrm{Target}\;\mathrm{Health}\;\mathrm{Expenditure}\;\mathrm{input}\;\left(i,\;t\right)}{\mathrm{Actual}\;\mathrm{Health}\;\mathrm{Expenditure}\;\mathrm{input}\;\left(i,\;t\right)}$$

(12) $$\mathrm{Birth}\;\mathrm{Rate}\;\mathrm{efficiency}\;=\frac{\mathrm{Actual}\;\mathrm{Birth}\;\mathrm{Rate}\;\mathrm{desirable}\;\mathrm{output}\;\left(i,\;t\right)}{\mathrm{Target}\;\mathrm{Birth}\;\mathrm{Rate}\;\mathrm{desirable}\;\mathrm{output}\;\left(i,\;t\right)}$$

(13) $$\mathrm{Respiratory}\;\mathrm{Diseases}\;\mathrm{efficiency}\;=\frac{\mathrm{Target}\;\mathrm{Respiratory}\;\mathrm{Diseases}\;\mathrm{Undesirable}\;\mathrm{output}\;\left(i,\;t\right)}{\mathrm{Actual}\;\mathrm{Respiratory}\;\mathrm{Diseases}\;\mathrm{Undesirable}\;\mathrm{output}\;\left(i,\;t\right)}$$

(14) $$\mathrm{Mortality}\;\mathrm{Rate}\;\mathrm{efficiency}\;=\frac{\mathrm{Target}\;\mathrm{Mortality}\;\mathrm{Rate}\;\mathrm{Undesirable}\;\mathrm{output}\;\left(i,\;t\right)}{\mathrm{Actual}\;\mathrm{Mortality}\;\mathrm{Rate}\;\mathrm{Undesirable}\;\mathrm{output}\;\left(i,\;t\right)}$$

(15) $${\mathrm{CO}}_{2}\;\mathrm{efficiency}\;=\frac{\mathrm{Target}\;{\mathrm{CO}}_{2}\;\mathrm{Undesirable}\;\mathrm{output}\;\left(i,\;t\right)}{\mathrm{Actual}\;{\mathrm{CO}}_{2}\;\mathrm{Undesirable}\;\mathrm{output}\;\left(i,\;t\right)}$$

(16) $$\mathrm{AQI}\;\mathrm{efficiency}\;=\frac{\mathrm{Target}\;\mathrm{AQI}\;\mathrm{Undesirable}\;\mathrm{output}\;\left(i,\;t\right)}{\mathrm{Actual}\;\mathrm{AQI}\;\mathrm{Undesirable}\;\mathrm{output}\;\left(i,\;t\right)}$$

If the target fixed assets, labor, energy consumption and health expenditure input equals the actual input, then the fixed assets, labor, energy consumption and health expenditure efficiencies equal 1, indicating overall efficiency. If the target fixed assets, labor, energy consumption and health expenditure input is less than the actual input, then the fixed assets, labor, energy consumption and health expenditure efficiencies are less than 1, indicating overall inefficiency.

If the target GDP and birth rate desirable output is equal to the actual GDP and birth rate desirable output, then the GDP and birth rate efficiency equals 1, indicating overall efficiency. If the actual GDP and birth rate desirable output is less than the target GDP and birth rate desirable output, then the GDP and birth rate efficiency is less than 1, indicating overall inefficiency.

If target respiratory diseases, mortality rate, CO_2_ and AQI undesirable output is equal to the actual respiratory diseases, mortality rate, CO_2_ and AQI undesirable output level, then respiratory diseases, mortality rate, CO_2_ and AQI efficiency equals 1, and is efficient. If target respiratory diseases, mortality rate, CO_2_ and AQI undesirable output is less than the actual respiratory diseases, mortality rate, CO_2_ and AQI undesirable output level, then Mortality Rate efficiency is less than 1, and is inefficient.

### 2.5. Data Sources and Description

**Figure 1:**
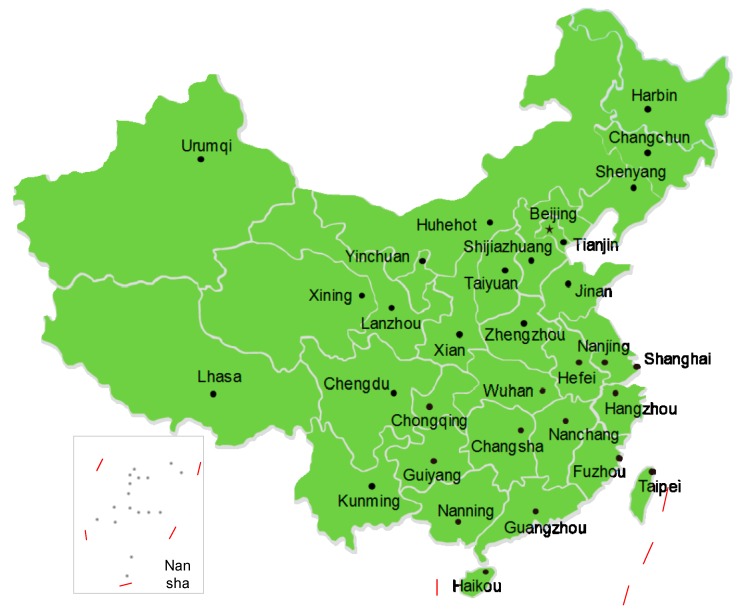
The thirty-one sample cities. (Source: the map is drawn by smart-draw software according to China’s National Administrative Planning Map [61]).

This study used panel data for 31 cities of the most developed cities from eastern China to western China. Economics and social development data from 2013 to 2016 were collected from the Statistical Yearbook of China [58], the *Demographics and Employment Statistical Yearbook of China*, and the *City Statistical Yearbooks* [59]. Air pollutant data were collected from the *China Environmental and Protection Bureau Annual Reports* and *China Environmental Statistical Yearbook* [60]. This study includes the 31 samples; Chengdu, Changsha, Chongqing, Guiyang, Hefei, Huhehot, Kunming, Lanzhou, Lhasa, Nanchang, Nanning, Taiyuan, Wuhan, Urumqi, Xian, Xining, Yinchuan, and Zhengzhou; Beijing, Changchun, Fuzhou, Guangzhou, Harbin, Haikou, Hangzhou, Jinan, Nanjing, Shanghai, Shenyang, Shijiazhuang, and Tianjin (see Figure 1). These 31 cities are all provincial capitals of China’s provinces and are distributed in various parts of the Central, East and West China. The core cities representing the economic and social development of China’s 31 administrative provinces/municipalities/regions are also the core areas of economic growth and industry of the provinces. Taking these 31 cities as the main research objects, they can represent the actual situation of economic, social and urban development in different regions of China, and the policy recommendations and measures proposed in this research can be benefit to the formulation and implementation of policies by the central and local governments.

**Figure 2:**
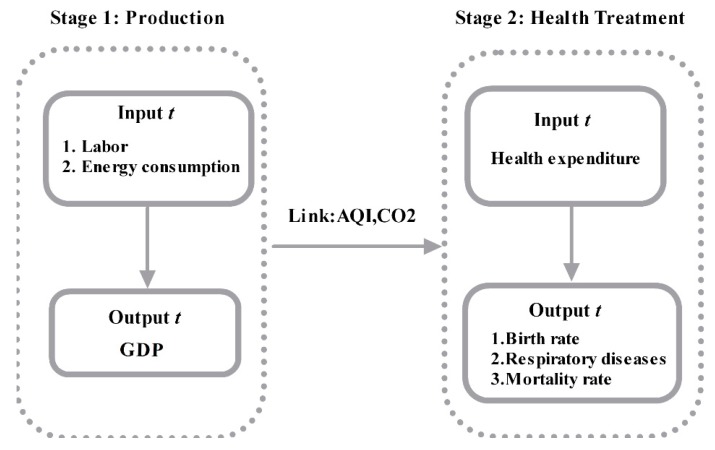
Modified undesirable EBM Two stage DEA.

Figure 2 shows the framework for the modified undesirable EBM Two-stage DEA Model for efficiency measurement and the variables.

The input and output variables are outlined in Table 1:

#### 2.5.1. Input Variables

Labor input (lab): employees in each city at the end of each year; unit: person. Fixed Assets (assets): fixed assets investment in each city; unit: 100 million CNY; Energy consumption (con): total energy consumption in each city; unit: 100 million Dun.

#### 2.5.2. Output Variable

Desirable output (GDP): GDP in each city; unit: 100 million CNY;
Production Stage and health stage link variables:
Carbon dioxide: CO_2_; a common greenhouse gas.

The Air quality Index (AQI) is the measured concentration of pollutants; particulate matter (PM_2.5_, PM_10_), sulfur dioxide (SO_2_), nitrogen dioxide (NO_2_), ozone (O_3_), and carbon monoxide (CO). PM_2.5_ and PM_10_ were taken as the 24-h average concentration, and the CO_2_ emissions data for each city were estimated from the energy consumption breakdown by fuel category.

The second stage: health stage
Input variables: Health Expenditure
Output variables: Birth rate, Respiratory Diseases, Mortality Rate

## 3. Results and Discussion

### 3.1. Input-Output Index Statistical Analyses

**Figure 3:**
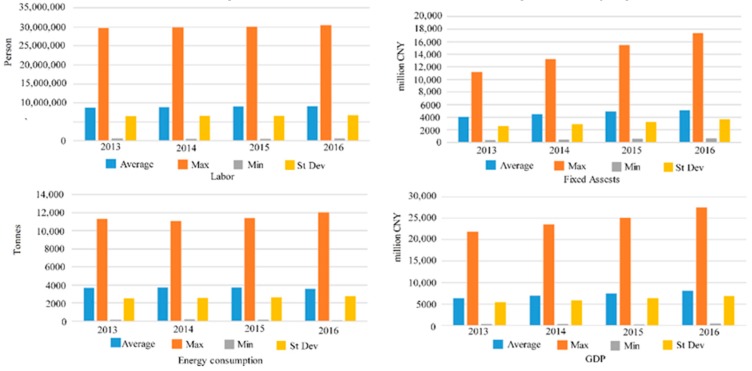
Labor, fixed assets, energy consumptions from 2013–2016.

Figure 3 shows the labor statistical analysis. From 2013 to 2016, the average value and the maximum value increased slowly, which was related to a decline in youth employment.

The statistical analysis for the fixed assets indicators shows that from 2013 to 2016, the fixed assets input grew rapidly, with the maximum value growing faster than the average value. There were differences in the fixed assets inputs between the cities.

The statistical analysis for the energy consumption indicators from 2013 to 2016 indicates that the maximum value increased in 2013, but declined in 2014, rose again to the level of 2013 in 2015, and increased again in 2016. The minimum value was the highest in 2014, declined in 2015 and further declined in 2016. There were significant energy consumption differences between the cities.

The GDP indicators from 2013 to 2016 illustrate that there was a significant rise in the maximum GDP value. However, because of the minimal increase in the minimum value, the average value increased at a slower rate than the maximum value. There were significant differences in the GDP growth between the cities.

**Figure 4:**
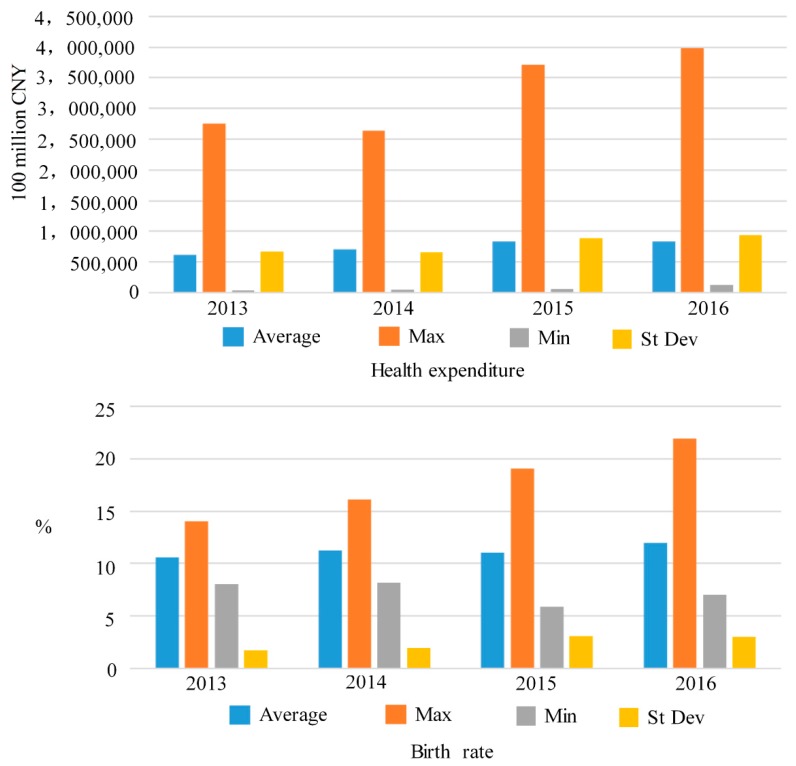
Health expenditures and birth rates from 2013–2016.

Figure 4 shows the health expenditure indicators and birth rate indicators from 2013 to 2016. As can be seen the government health expenditure input maximum value increased significantly in 2014 and 2015. There was also a large increase in the minimum value, especially in 2016 when the government increased investment.

The birth rate indicators from 2013 to 2016 from 2013 to 2016 show that the maximum value rose sharply, however, the minimum value first declined to the lowest level in in 2015, but rose again in 2016.

### 3.2. Total City Efficiency Scores for Each Year

In this paper, we assess the relative efficiency of decision making units based on a undesirable EBM Two-stage DEA method. Table 2 shows the overall efficiency scores for each city from 2013 to 2016. As can be seen, there were significant differences between the cities, with the efficiency scores changing markedly each year.

Of the 31 cities, only Guangzhou and Shanghai had efficiency scores of 1 in all four years, Lhasa and Nanning had efficiency scores of 1 in the first three years, but then fell to 0.86 in 2016. Beijing’s efficiency score was 1 in 2013 and 2015 but it declined slightly in 2014 and 2016.

As can be seen in Figure 4, only Kunming had an overall efficiency score that continued to rise across all four years, Chongqing and Guiyang has four-year upward fluctuations, and Changchun and Zhengzhou has increasing overall efficiency in the first three years but declined in the last year. Generally, this analysis indicated that the overall efficiency improvements in most cities was poor, with the total efficiency in only Chongqing and Guiyang improving over the four years.

Huhehot, Nanchang, Nanning, Tianjin, Wuhan, and Xian all had continuous decreases in overall efficiency across the four years; therefore, the need for improvement was expanding. Xining, Yinchuan, Changsha, Jinan, Shenyang, Lanzhou, Shijiazhuang, and Taiyuan had rising overall efficiencies in the first three years but suffered declines in 2016.

### 3.3. Comparison of the Radial and Non-Radial Non-Efficiency Scores in Each City

As shown in Table 3, the Epsilon scores for the inputs and outputs were all below 0.5, indicating that all data were suitable for the radial input analysis of the efficiency evaluation.

Our research analyzes the efficiency of various indicators including: Fixed assets, Labor, energy consumption, GDP, Health Expenditure, Birth Rate, Respiratory Diseases, Mortality Rate, CO_2_ and AQI related to energy consumption and environmental pollution and inputs in government medical treatment. On the other hand, this paper evaluates and compares the input and output efficiency of local governments in energy, environment and health management, and analyze the room for improvement of resource utilization to improve the efficiency and fairness of resource allocation between central and local governments.

### 3.4. 2013 to 2016 Efficiency Scores and Rankings for Labor, Fixed Assets, and Energy Consumption

As shown in Table 4, Guangzhou, Lhasa, Nanning and Shanghai had labor efficiencies of 1; therefore, there was no need for further improvement.

However, only Guiyang and Haikou has improving labor efficiency scores from 2013 to 2016. Chengdu, Chongqing, Harbin, Nanchang, Shijiazhuang, Tianjin, Wuhan, Xian, Yinchuan, Zhengzhou all had falling labor efficiencies, indicating that more improvements were necessary. The labor efficiencies in some other cities were fluctuating but fell in 2016.

Guangzhou, Lhasa, and Shanghai all had fixed assets efficiency scores of 1 from 2013 to 2016; therefore, there was no further need for improvements. Hefei, Huhehot, Nanjing, Xian and Shenyang all had rising fixed assets efficiencies; however, the other 23 cities had declining or fluctuating fixed asset indicators. Nanning had a fixed asset efficiency of 1 in the first three years; however, it fell to around 0.59 in 2016, and there was a significant need for improvement.

From 2013 to 2016, the energy consumption efficiencies in Guiyang, Lanzhou and Hangzhou continued to increase. Chongqing, Fuzhou, Haikou, Hangzhou, Hefei, Huhehot, Jinan, Kunming, Nanjing, Shenyang, Tianjin, Xining, and Urumqi had upwardly fluctuating energy consumption efficiencies, with Jinan and Shenyang having larger increases; Jinan’s efficiency score increased from 0.5 in 2013 to 1 in 2016, and Shenyang’s rose from 0.6 in 2013 to 1 in 2016.

The energy consumption efficiencies in Harbin, Nanchang, Nanning, Xian, and Yinchuan continued to decline, and the energy efficiencies in the other 10 cities had downward fluctuating trends. Except for eight cities, the fixed assets and employment efficiency scores were declining of had downward fluctuating trends.

### 3.5. 2013 to 2016 Health Expenditure, GDP, and Birth Rate Efficiency Scores and Rankings

Beijing, Wuhan, Xining, and Nanning government health expenditure input indicators dropped significantly; however, in most other cities, health expenditure efficiency had a continuous increase or upward fluctuating trends, indicating that the need for improvements was shrinking. Changsha, Chengdu, Chongqing, Guiyang, Hangzhou, Hefei, Jinan, Nanjing and Tianjin all had larger efficiency increases, with Tianjin’s efficiency rising from 0.1 in 2013 to around 0.7 in 2016, Jinan’s rising from 0.3 in 2013 to 1 in 2016, and Hefei’s rising from 0.43 in 2013 to 1 in 2016. Changchun, Harbin, Huhehot, Kunming, Nanchang, Shenyang, Shijiazhuang, Taiyuan, Xian, and Zhengzhou had lower efficiency rises than the above nine cities.

There were less cities with higher GDP efficiencies that cities with rising GDP efficiencies. Chongqing, Guiyang, Hangzhou, Jinan, Kunming and Shenyang had rising GDP efficiencies; however, Changchun, Fuzhou, Harbin, Haikou, Hefei, Huhehot, Nanchang, Nanning, Wuhan, Urumqi, and Xian all experienced significant GDP efficiency declines, indicating that the need for GDP efficiency improvements was expanding.

Fuzhou, Guangzhou, Haikou, Lhasa, Nanning, and Shanghai all had birth rate efficiencies of 1 for all four years from 2013–2016; however, Beijing, Changchun, Shenyang, Kunming, Lanzhou, Shijiazhuang, and Wuhan had continuous downward fluctuations. The birth rate efficiencies in the other 28 cities continued to rise or had an upward fluctuation, with Changsha, Hefei, Jinan, and Shenyang experiencing larger increases (Table 5).

### 3.6. 2013 to 2016 Respiratory Diseases and Mortality Rate Efficiency Scores and Rankings

Table 6 shows that Beijing, Changchun, Guiyang, Harbin, Kunming, Lanzhou, Shijiazhuang, Tianjin, Wuhan, and Zhengzhou all had decreasing respiratory disease efficiencies, with Beijing, Zhengzhou, and Wuhan experiencing the largest falls, with Beijing, falling from 1 in 2013 to 0.82 in 2016, and Wuhan falling from 1 in 2013 to 0.84 in 2016. However, in the other cities, the respiratory diseases efficiencies were rising, with Changsha, Chongqing, Hefei, and Jinan experiencing larger increases. Hefei and Jinan’s respiratory diseases efficiencies rose from around 0.65 in 2013 to 1 in 2016, and Changsha’s rose from 0.6 in 2013 to 0.94 in 2016.

There were large differences in the mortality rate efficiency improvements between the cities from 2013 to 2016. Fuzhou, Guangzhou, Haikou, Lhasa, and Shanghai all had mortality rate efficiencies of 1, and Changsha, Chongqing, Hefei, Hangzhou, Jinan, and Nanjing had large increases; Hefei and Jinan’s mortality rate efficiencies rose from 0.61 in 2013 to 1 in 2016, Hangzhou’s rose from 0.74 in 2012 to 0.93 in 2016, and Nanjing’s rose from 0.69 in 2013 to 0.91 in 2016.

A further 15 cities had upward mortality rate efficiency trends but there were 11 cities with reduced mortality rate efficiencies. Overall, there were more cities with improved mortality rate efficiencies than cities with reduced efficiencies.

### 3.7. 2013 to 2016 CO2 and AQI Efficiency Scores and Rankings

As shown in Table 7, Fuzhou, Guangzhou, Haikou, Lhasa, and Shanghai all achieved carbon dioxide and AQI emissions efficiency scores of 1, indicating that there was no need for further carbon emissions and air pollution emissions improvements. Zhengzhou, Yinchuan, Xian, Tianjin, Wuhan, Shijiazhuang, Nanning, Nanchang, Huhehot, Harbin and Changchun all had large fluctuations with falling CO_2_ emissions efficiencies, of which Harbin’s fell the most from around 0.9 in 2013 to around 0.73 in 2016.

The carbon emissions efficiencies in the other 15 cities all increased over the four years, with Jinan, Shenyang and Xining experiencing large improvements; Jinan’s rose from 0.56 in 2013 to 1 in 2016, Shenyang’s rose from 0.67 in 2013 to 1 in 2016, and Xining’s rose from 0.27 in 2013 to 0.56 in 2016.

Chongqing, Guangzhou, Fuzhou, Haikou, Nanning, Shanghai, and Lhasa had AQI emissions efficiency of 1 in all four years, and therefore there were no further improvements needed. Twelve cities had downward AQI emissions efficiency trends; Shijiazhuang’s dropped from 0.82 in 2013 to 0.49 in 2016, Xining’s dropped from 0.74 in 2013 to 0.48 in 2016, Nanchang’s fluctuated from 1 in 2013 to 0.83 in 2016, and Nanjing’s dropped from 0.936 in 2013 to 0.778 in 2016.

The AQI emissions efficiencies in Jinan, Shenyang and Urumqi fluctuated significantly, with all having efficiencies of 1 in 2013, 2014 and 2016; however, in 2015, the AQI emissions efficiencies dropped significantly to around 0.40.

### 3.8. Comparison of Undesirable Output Efficiency and Main Policy

From Table 8, Chengdu, Hangzhou, Hefei, Huhehot, Jinan, Lanzhou, Nanchang, Nanjing, Shenyang, Shijiazhuang, Urumqi, Yinchuan should address on the emission control of carbon dioxide and air pollutants. These cities can adjust the industrial structure and develop tertiary industries and new energy, and reduce emissions of air pollutants through the control of pollution sources such as vehicle and living air pollutants emissions.

Beijing, Changchun, Wuhan, Xian, Xining, Zhengzhou should strengthen the control of air pollutant emissions, especially on carbon dioxide emissions. The measures on industrial restructuring, industrial transformation, and energy transformation should be taken. These cities should also develop public transport, reduce air pollution emissions and use new energy and clean fossil energy to reduce the damage caused by air pollution to population health.

Changsha, Chongqing, Guiyang, Harbin, Kunming, Nanning, Taiyuan, Tianjin should focus on the management and control of carbon dioxide emissions. The above cities have better meteorological conditions, and the efficiency of air pollutants is slightly better than that of carbon dioxide emission. Therefore, in policy measures, carbon dioxide emission reduction should be priority. In particular, cities such as Taiyuan and Tianjin can achieve carbon reduction targets through industrial transformation and new energy and the reform on traditional energy use. In the meanwhile, other air pollutants emission should be reduced.

Respiratory disease efficiency and mortality efficiency are improved in most cities. Beijing, Changchun, Guiyang, Harbin, Kunming, Shenyang Shijiazhuang, Tianjin should focus on respiratory diseases and mortality and population health. Fuzhou, Guangzhou, Haikou, Lhasa, Shanghai should maintain the existing environmental efficiency and be a model.

## 4. Conclusions and Policy Recommendation

This research used a two-stage network model and undesirable output to assess the urban environmental efficiencies and health efficiencies in 31 Chinese cities from 2013 to 2016, the results from which were as follows:

(1) Of the 31 cities, only Lhasa, Guangzhou, and Shanghai had overall efficiency scores of 1 for all four years. Nanning’s total efficiency score was 1 from 2013 to 2015 but fluctuated down in 2016. The overall efficiency in Beijing was 1 in 2013 and 2015, 0.94 in 2014, and 0.84 in 2016, indicating that the need for improvements in Beijing was expanding, which was also true for most other cities.
(2) Compared with the number of cities with increasing overall efficiency scores, most cities had decreasing overall efficiency scores; therefore, the improvements in overall efficiencies were not optimistic.
(3) Health expenditure efficiency in most cities was rising except for the input efficiencies in Beijing, Wuhan, Xining, and Nanning, all of which dropped significantly.
(4) Except for the cities that had GDP efficiencies of 1, there were less cities with rising GDP efficiencies (6) than cities with decreasing GDP efficiencies (11); therefore, the need for GDP efficiency improvements was expanding.
(5) The birth rate efficiency scores in Fuzhou, Guangzhou, Haikou, Lhasa, Nanning, and Shanghai were all 1, 7 cities had downward trends while the other 28 cities had upward trends; therefore, in general, this indicator was improving in most cities.
(6) The respiratory diseases treatment efficiencies decreased in 10 cities; however, in all other cities it was rising, and the need for improvements was significantly reducing.
(7) Five cities had mortality rate efficiencies of 1 and the efficiency increased significantly in 15 other cities; however, the mortality rate efficiencies declined in 11 cities.
(8) The carbon emissions efficiencies rose in most cities.

The results indicate that there was still a significant need for improvement in most cities. Therefore, it is necessary to strengthen investments in treatments, and more specific measures should be taken to effectively to increase urban pollution and health treatment efficiencies. Based on the above research conclusions, this study gives the following policy recommendations:

(1) Carbon dioxide emissions and air pollutants have brought great challenges to the environmental governance of central and local governments. The impact of air pollutants on human health is large and long-lasting and should be prioritized in governance work. Even the air pollutant efficiency of some cities is better than the carbon dioxide emissions, the reduction of air pollutants cannot be overlooked. Chengdu, Hangzhou, Hefei, Huhehot, Jinan, Lanzhou, Nanchang, Nanjing, Shenyang, Shijiazhuang, Urumqi, Yinchuan should be more effective in controlling carbon dioxide emissions and air pollution emissions, using industrial transformation, and energy transformation to achieve emission reduction. Beijing, Changchun, Wuhan, Xian, Xining, Zhengzhou should control air pollutants first and then consider carbon dioxide emissions reduction. Changsha, Chongqing, Guiyang, Harbin, Kunming, Nanning, Taiyuan, Tianjin, should control carbon dioxide emissions first and then consider the treatment and control of air pollutants. Fuzhou, Guangzhou, Haikou, Lhasa, Shanghai should maintain better environmental efficiency and continue to improve air pollutants and carbon dioxide emissions.
(2) Because China has a vast territory, there are very large differences in economic and social development. The economy in most regions in the east is significantly better than in the central and western regions. Therefore, the efficiency scores in most eastern cities (Beijing, Shanghai, Guangzhou) were at a higher level and there was less need for improvement. With rapid economic development being at the cost of increased energy consumption and environmental pollution, it is necessary to use comprehensive treatments and increase the sustainable development of the cities. As Beijing is the capital, it attracts significant government investment. However, the treatment efficiency was not high and was on a downward trend. Urban development consumes a great deal of energy that generates pollution, which had led to a significant decline in environmental efficiency; therefore, the treatments associated with respiratory diseases, air pollution, and carbon emissions should be strengthened.
(3) For inland cities, such as Chengdu and Chongqing, the efficiency scores in 2016 were significantly higher than in 2014 and 2015, indicating that the treatment effects were improving the overall environment. Compared with the coastal cities, many mid-western cities had lower efficiency scores, with many experiencing significant declines in treatment efficiencies in 2016. Therefore, while investment in many cities in 2016 increased, the effects have not yet been realized and it is still necessary to reduce excessive investment and resource waste.
(4) Environmental pollution in cities is associated with respiratory diseases, which was measured in terms of the respiratory disease treatment and mortality rate efficiencies. Environmental pollution problems should be solved from the source and treatment efficiency increased. Also, Beijing, Changchun, Guiyang, Harbin, Kunming, Shenyang Shijiazhuang, Tianjin should pay attention to respiratory diseases and mortality, fund improvements to the quality of medical equipment and reduce disease.

## Figures and Tables

**Table 1:** Input and output variables.

	Input Variables	Output Variables	Link
Stage 1	Labor (Lab)	GDP	AQI
	Fixed assets (asset)		CO_2_
	Energy consumption (con)		
Stage 2	Health Expenditure	Birth rate	
		Respiratory Diseases;	
		Mortality Rate	

**Table 2:** Overall efficiency scores for each city from 2013–2016.

NO	DMU	2013	2014	2015	2016
1	Beijing	1	0.989601	1	0.849723
2	Changchun	0.815132	0.827072	0.827642	0.608184
3	Changsha	0.89342	0.878934	0.863749	0.902829
4	Chengdu	0.642234	0.608838	0.66257	0.560853
5	Chongqing	0.581473	0.594271	0.589402	0.707737
6	Fuzhou	0.964131	0.621109	0.934035	0.965527
7	Guangzhou	1	1	1	1
8	Guiyang	0.457578	0.485777	0.488281	0.599234
9	Harbin	0.808949	0.79447	0.796574	0.502568
10	Haikou	0.948872	0.669205	0.928245	0.948917
11	Hangzhou	0.843423	0.833258	0.833978	0.843787
12	Hefei	0.77078	0.777052	0.741251	0.97223
13	Huhhot	0.873322	0.852322	0.790979	0.754991
14	Jinan	0.760292	0.725372	0.653882	1
15	Kunming	0.453256	0.458886	0.50625	0.591795
16	Lanzhou	0.525941	0.457756	0.453991	0.682741
17	Lhasa	1	1	1	1
18	Nanchang	0.868581	0.84167	0.801384	0.694567
19	Nanjing	0.8851	0.846445	0.888145	0.802205
20	Nanning	1	1	1	0.865003
21	Shanghai	1	1	1	1
22	Shenyang	0.725947	0.686892	0.661513	0.860712
23	Shijiazhuang	0.456604	0.425277	0.384795	0.459909
24	Taiyuan	0.58708	0.49415	0.493603	0.63538
25	Tianjin	0.823033	0.823165	0.788025	0.664835
26	Wuhan	0.950552	0.789078	0.771077	0.766357
27	Urumqi	0.916579	0.893509	0.594525	0.984552
28	Xian	0.654069	0.65707	0.615445	0.598831
29	Xining	0.508144	0.435087	0.428298	0.590434
30	Yinchuan	0.646088	0.589195	0.572028	0.767119
31	Zhengzhou	0.904107	0.914056	0.928053	0.706123

**Table 3:** Epsilon Scores from 2013–2016.

	2013	2014	2015	2016
Epsilon for EBMX	0.057	0.051	0.095	0.1119
Epsilon for y	0.3594	0.2465	0.242	0.2667

**Table 4:** Employment, fixed assets investment, and energy consumption efficiency scores.

No.	DMU	2013 Em	2014 Em	2015 Em	2016 Em	2013 Asset	2014 Asset	2015 Asset	2016 Asset	2013 Com	2014 Com	2015 Com	2016 Com
1	Beijing	1	0.880763374	1	0.8524	1	0.9592901	1	0.9210143	1	0.99542	1	0.99856
2	Changchun	0.8991	0.90611047	0.90711	0.8057	0.7215939	0.7411042	0.744306	0.6096182	0.8991	0.90611	0.907108	0.80574
3	Changsha	0.94709	0.938663441	0.93327	0.952	0.5721516	0.5235194	0.460713	0.4841279	0.65715	0.66672	0.664614	0.65718
4	Chengdu	0.78317	0.757419183	0.7979	0.7446	0.6087696	0.6515081	r	0.6197391	0.78317	0.75742	0.797897	0.74458
5	Chongqing	0.63104	0.500024235	0.52621	0.5274	0.5582939	0.4286548	0.421291	0.3855462	0.7371	0.74854	0.747195	0.75362
6	Fuzhou	1	0.733257885	1	1	0.8617823	0.5204487	0.641657	0.6194099	1	0.76774	1	1
7	Guangzhou	1	1	1	1	1	1	1	1	1	1	1	1
8	Guiyang	0.62894	0.654998838	0.65875	0.6882	0.6146963	0.5623476	0.485601	0.4681071	0.47867	0.55585	0.591906	0.61108
9	Harbin	0.89606	0.885502631	0.88703	0.7242	0.5813495	0.8774743	0.857175	0.6756626	0.89606	0.8855	0.887034	0.73003
10	Haikou	0.55735	0.586010339	0.96348	0.9998	0.9758649	0.8029476	1	0.8938472	0.97586	0.80295	1	1.000
11	Hangzhou	0.91704	0.910943901	0.91314	0.9319	0.7152775	0.6503048	0.608121	0.6141956	0.73959	0.76052	0.798248	0.80345
12	Hefei	0.87244	0.876119794	0.85484	1	0.5259938	0.5418133	0.477602	0.5705747	0.87244	0.87612	0.85484	1
13	Huhehot	0.93601	0.921804653	0.88594	0.9181	0.7780742	0.756976	0.753144	0.8292995	0.38497	0.75263	0.724236	0.69614
14	Jinan	0.86547	0.84230184	0.79403	1	0.8654708	0.8423018	0.695281	1	0.56354	0.54639	0.554859	1
15	Kunming	0.62511	0.630134621	0.67327	0.6673	0.5159696	0.544736	0.571462	0.5270656	0.53329	0.53631	0.673272	0.66728
16	Lanzhou	0.69119	0.62970179	0.62858	0.6486	0.6911945	0.6297018	0.607536	0.5982818	0.39525	0.34542	0.284083	0.31471
17	Lhasa	1	1	1	1	1	1	1	1	1	1	1	1
18	Nanchang	0.93126	0.915454664	0.89282	0.8131	0.6223186	0.6027086	0.543299	0.4671772	0.93126	0.91545	0.892823	0.81313
19	Nanjing	0.94253	0.920028875	0.94426	0.9628	0.5596182	0.5317071	0.588637	0.6139003	0.64366	0.61318	0.884983	0.86169
20	Nanning	1	1	1	0.6126	1	1	1	0.5876837	1	1	1	0.87547
21	Shanghai	1	1	1	1	1	1	1	1	1	1	1	1
22	Shenyang	0.84477	0.817273553	0.80052	1	0.4111169	0.4090846	0.52199	1	0.64117	0.64932	0.63962	1
23	Shijiazhuang	0.62809	0.597933513	0.55923	0.576	0.6155488	0.5774181	0.440956	0.4641232	0.4664	0.42375	0.378611	0.38869
24	Taiyuan	0.74324	0.664259912	0.66666	0.6827	0.7432409	0.6642599	0.642631	0.6826506	0.17809	0.17449	0.168238	0.20609
25	Tianjin	0.90639	0.906020087	0.8873	0.9113	0.4624992	0.4587871	0.420641	0.4033572	0.69375	0.70324	0.700194	0.67872
26	Wuhan	0.976	0.884533161	0.87519	0.8883	0.7022254	0.5266488	0.497459	0.559047	0.976	0.7285	0.760198	0.74618
27	Urumqi	0.95647	0.904900248	0.74691	0.9753	0.956474	0.943938	0.701972	1	0.95647	0.94394	0.672827	1
28	Xian	0.79259	0.794542591	0.76402	0.733	0.4964975	0.4999415	0.553458	0.585522	0.79259	0.79454	0.764021	0.73304
29	Xining	0.67649	0.608688283	0.60402	0.6183	0.6764852	0.5877194	0.588659	0.5693241	0.2649	0.23926	0.244148	0.56395
30	Yinchuan	0.78832	0.744749892	0.7347	0.75	0.6361415	0.547706	0.522813	0.50217	0.37095	0.33598	0.272031	0.2703
31	Zhengzhou	0.95091	0.956111727	0.96513	0.8493	0.7003059	0.7250648	0.667886	0.5763073	0.95091	0.95611	0.965132	0.84935

**Table 5:** Health expenditure, GDP, and birth rate.

No.	DMU	2013 Gov	2014 Gov	2015 Gov	2016 Gov	2013 GDP	2014 GDP	2015 GDP	2016 GDP	2013 Birth	2014 Birth	2015 Birth	2016 Birth
1	Beijing	1	0.96094	0.05671	0.5166	1	0.995465	1	0.99857	1	0.963767	0.789418	0.88558
2	Changchun	0.25316	0.46671	0.47856	0.3657	0.91604	0.920954	0.92166	0.86009	0.852579	0.82427	0.789224	0.82091
3	Changsha	0.59978	0.94384	0.93633	0.9537	0.95215	0.945366	0.94113	0.9562	0.77771	0.949508	0.943526	0.95765
4	Chengdu	0.24204	0.78878	0.77694	0.6993	0.84876	0.836664	0.85607	0.83094	0.789178	0.851511	0.845755	0.81222
5	Chongqing	0.26016	0.47816	0.22884	0.9113	0.8277	0.832685	0.83209	0.83495	0.856535	0.852643	0.827296	0.92465
6	Fuzhou	1	1	1	1	0.82228	0.841409	0.70848	0.71394	1	1	1	1
7	Guangzhou	1	1	1	1	1	1	1	1	1	1	1	1
8	Guiyang	0.48762	0.61125	0.77832	0.814	0.78701	0.795858	0.79718	0.80797	0.873615	0.871849	0.846413	0.86444
9	Harbin	0.25863	0.3673	0.34956	0.3512	0.91395	0.906837	0.90785	0.82469	0.7861	0.7448	0.750149	0.78346
10	Haikou	1	1	1	1	0.97698	0.858653	0.6879	0.6565	1	1	1	1
11	Hangzhou	0.27926	0.87943	0.74457	0.9093	0.92885	0.924408	0.92599	0.94004	0.828756	0.902858	0.830937	0.92326
12	Hefei	0.43444	0.43593	0.84694	1	0.89837	0.900718	0.8875	0.74737	0.798231	0.775016	0.904677	1
13	Huhehot	0.67207	0.78388	0.66767	0.8061	0.94327	0.93238	0.90713	0.9296	0.854945	0.849102	0.805043	0.86027
14	Jinan	0.31353	0.52193	0.74403	1	0.89399	0.880114	0.85412	1	0.7962	0.802036	0.830702	1
15	Kunming	0.68631	0.84896	0.77153	0.7134	0.78575	0.787401	0.8024	0.80022	0.975447	0.884002	0.843185	0.87089
16	Lanzhou	0.62664	0.84152	0.78017	0.666	0.8091	0.787258	0.78689	0.79364	1	1	0.847304	0.98908
17	Lhasa	1	1	1	1	1	1	1	1	1	1	1	1
18	Nanchang	0.41275	0.50408	0.59055	0.5141	0.93957	0.927683	0.91174	0.86397	0.869366	0.875546	0.846009	0.879
19	Nanjing	0.29595	0.88139	0.72304	0.8249	0.94845	0.931056	0.94985	0.96537	0.807256	0.904131	0.821769	0.87031
20	Nanning	1	1	1	0.5744	1	1	1	0.86983	1	1	1	1
21	Shanghai	1	1	1	1	1	1	1	1	1	1	1	1
22	Shenyang	0.56643	0.71526	0.49546	0.8638	0.88155	0.866179	0.85741	1	0.767789	0.818579	0.748871	0.89298
23	Shijiazhuang	0.35585	0.37972	0.7867	0.6887	0.78673	0.777141	0.76574	0.77057	0.813034	0.821527	0.850482	0.80813
24	Taiyuan	0.73754	0.7174	0.79461	0.877	0.83036	0.799136	0.8	0.80587	0.860941	0.885525	0.854415	0.9013
25	Tianjin	0.0753	0.55627	0.45309	0.6951	0.92116	0.92089	0.90803	0.92469	0.814586	0.793887	0.794337	0.81061
26	Wuhan	1	0.72251	0.58542	0.8523	0.9771	0.906196	0.90012	0.9087	1	0.87223	0.880181	0.88597
27	Urumqi	1	1	0.95092	1	0.95996	0.94959	0.83197	0.80415	1	1	0.95531	1
28	Xian	0.25664	0.37726	0.68999	0.626	0.8534	0.85438	0.83968	0.82596	0.804754	0.88843	0.828994	0.84331
29	Xining	0.87413	0.93019	0.83717	0.6702	0.80358	0.780485	0.77902	0.78355	0.899443	0.945976	0.87717	0.89988
30	Yinchuan	0.97799	0.94424	0.89527	0.9838	0.85128	0.831016	0.82667	0.83332	0.987265	0.988725	0.913409	0.99467
31	Zhengzhou	0.20557	0.27189	0.4091	0.3529	0.9553	0.959653	0.9674	0.88423	0.867986	0.863565	0.891459	0.87214

**Table 6:** Respiratory disease mortality efficiency scores.

No.	DMU	2013 Respiratory Diseases	2014 Respiratory Diseases	2015 Respiratory Diseases	2016 Respiratory Diseases	2013 Mort	2014 Mort	2015 Mort	2015 Mort
1	Beijing	1	0.9351834	0.7547981	0.8173057	1	0.960936	0.780715	0.851629
2	Changchun	0.7845439	0.7290381	0.6356186	0.7209566	0.790939	0.729038	0.635619	0.720957
3	Changsha	0.5985419	0.943837	0.9363347	0.93773943	0.599781	0.943837	0.936335	0.953731
4	Chengdu	0.353543	0.5248746	0.5263787	0.40725183	0.635481	0.788785	0.776945	0.699285
5	Chongqing	0.7850896	0.7910674	0.7361652	0.87849315	0.798806	0.791067	0.736165	0.91128
6	Fuzhou	1	1	1	1	1	1	1	1
7	Guangzhou	1	1	1	1	1	1	1	1
8	Guiyang	0.8285099	0.8276835	0.7783176	0.81401447	0.830862	0.827684	0.778318	0.814014
9	Harbin	0.6223008	0.4787582	0.5005972	0.61802974	0.62618	0.478758	0.496996	0.61803
10	Haikou	1	1	1	1	1	1	1	1
11	Hangzhou	0.5968402	0.8078024	0.6509197	0.63179293	0.739557	0.879434	0.744569	0.909349
12	Hefei	0.6587714	0.590962	0.8822236	1	0.661724	0.590962	0.882224	1
13	Huhehot	0.7929696	0.7838774	0.6804446	0.80607653	0.795665	0.783877	0.680445	0.806076
14	Jinan	0.651048	0.6722845	0.7440329	1	0.655975	0.672285	0.744033	1
15	Kunming	0.8505386	0.8489619	0.7715309	0.78514154	0.974179	0.848962	0.771531	0.825943
16	Lanzhou	0.999649	1	0.7801701	0.98883699	1	1	0.78017	0.988837
17	Lhasa	1	1	1	1	1	1	1	1
18	Nanchang	0.8216687	0.8343026	0.7774755	0.84036978	0.823165	0.834303	0.777475	0.84037
19	Nanjing	0.6809365	0.881388	0.7230441	0.70638892	0.686346	0.881388	0.723044	0.824899
20	Nanning	1	1	1	1	1	1	1	0.987957
21	Shanghai	1	1	1	1	1	1	1	1
22	Shenyang	0.5664307	0.7152643	0.4954621	0.56899178	0.524542	0.629656	0.436969	0.557693
23	Shijiazhuang	0.6946655	0.7224598	0.7866965	0.64157819	0.701364	0.72246	0.786697	0.688659
24	Taiyuan	0.8035407	0.8515336	0.7946128	0.87701927	0.807366	0.851534	0.794611	0.877019
25	Tianjin	0.6923424	0.6493323	0.6506331	0.6523241	0.705305	0.649332	0.650633	0.695142
26	Wuhan	1	0.8283719	0.8424187	0.84428669	1	0.803032	0.842419	0.852283
27	Urumqi	1	1	0.8202437	1	1	1	0.950923	1
28	Xian	0.6739938	0.8563826	0.7401074	0.76480002	0.679666	0.577883	0.740107	0.771797
29	Xining	0.8740992	0.939432	0.8371692	0.87480972	0.874129	0.939432	0.83714	0.86864
30	Yinchuan	0.9857324	0.9884652	0.8952715	0.99461062	0.986932	0.988465	0.895263	0.992445
31	Zhengzhou	0.8206256	0.8123653	0.8613643	0.61338284	0.769943	0.730502	0.861364	0.828208

**Table 7:** 2013–2016 AQI treatment and carbon dioxide emission treatment efficiency scores.

No.	DMU	2013 CO_2_	2014 CO_2_	2015 CO_2_	2016 CO_2_	2013 AQI	2014 AQI	2015 AQI	2016 AQI
1	Beijing	1	0.987029	1	0.99856	1	1	1	0.911188
2	Changchun	0.93627	0.872525	0.90711	0.80574	1	1	0.51444	0.787896
3	Changsha	0.66249	0.662893	0.66461	0.65718	1	1	0.66639	1
4	Chengdu	0.7123	0.830107	0.7979	0.74458	1	1	0.72416	0.85539
5	Chongqing	0.70269	0.772805	0.74719	0.75362	1	1	1	1
6	Fuzhou	1	1	1	1	1	1	1	1
7	Guangzhou	1	1	1	1	1	1	1	1
8	Guiyang	0.53085	0.504128	0.73552	0.61108	1	1	0.65398	1
9	Harbin	0.90319	0.875912	0.88703	0.73003	0.67555	1	0.65821	1
10	Haikou	1	1	1	1	1	1	1	1
11	Hangzhou	0.75835	0.744039	0.79825	0.80345	1	1	0.57907	0.722116
12	Hefei	0.87818	0.872797	0.85484	1	0.75795	1	0.4995	1
13	Huhehot	0.75483	0.384172	0.72424	0.69614	1	0.87452	0.41833	1
14	Jinan	0.55995	0.546146	0.55486	1	1	1	0.40835	1
15	Kunming	0.53348	0.535843	0.67327	0.66728	1	1	0.92484	1
16	Lanzhou	0.38517	0.347773	0.28408	0.31471	0.78338	0.55009	0.43372	0.471312
17	Lhasa	1	1	1	1	1	1	1	1
18	Nanchang	0.9182	0.93236	0.89282	0.81313	1	1	0.68149	0.829057
19	Nanjing	0.61562	0.645276	0.88498	0.86169	0.9357	1	0.62126	0.778348
20	Nanning	1	1	1	0.87547	1	1	1	1
21	Shanghai	1	1	1	1	1	1	1	1
22	Shenyang	0.66545	0.627728	0.63962	1	1	1	0.48321	1
23	Shijiazhuang	0.50009	0.3922	0.59981	0.38869	0.82022	1	0.39356	0.487675
24	Taiyuan	0.19052	0.163025	0.16824	0.20609	0.96204	0.41809	0.40877	0.41523
25	Tianjin	0.69272	0.703578	0.70019	0.67872	0.93238	1	0.5781	0.655972
26	Wuhan	1	0.749974	0.7602	0.74618	1	1	0.62439	0.497028
27	Urumqi	1	1	0.67283	1	1	1	0.33052	1
28	Xian	0.77568	0.812061	0.76402	0.73304	0.74055	1	0.43468	0.654921
29	Xining	0.26656	0.233276	0.24415	0.56395	0.74043	0.49459	0.50091	0.480425
30	Yinchuan	0.33957	0.369273	0.27203	0.2703	0.74327	0.57298	0.41242	0.438677
31	Zhengzhou	0.95579	0.950753	0.96513	0.84935	0.87642	1	0.49866	0.749967

**Table 8:** Comparison of undesirable output efficiency and policy priorities of each city.

DMU	Respiratory Disease and Mortality Efficiency Score	CO and AQI Efficiency Score	Main Policy
Beijing	Respiratory disease efficiency and mortality efficiency continue to decline	The efficiency score of CO_2_ is slightly up and down, and the minimum is about 0.99. The room for improvement is very small. AQI is 1 in the first three years, but it declines slightly in the last year. AQI efficiency is slightly lower than CO_2_ in the last year.	There is small room for improvement in efficiency of carbon emissions and air pollution. Air pollution is more concerned in Beijing.
Changchun	Respiratory disease efficiency and mortality efficiency decline slightly	CO_2_ efficiency score decreases, and drops to around 0.81 in the last year. The AQI efficiency score drops from 1 in the previous two years to 0.51 in 2015. Then rises to 0.79, the room for improvement in the last two years is greater than CO_2_.	Air pollution treatment should be more important than carbon emissions.
Changsha	Respiratory disease efficiency and mortality efficiency have risen dramatically.	Carbon dioxide efficiency has not changed much, staying around 0.66. AQI efficiency score is better than CO2, only the efficiency score in 2015 is about 0.67, and other years are 1.	Carbon emission treatment should be prior to air pollutants.
Chengdu	The increase in respiratory disease efficiency and mortality efficiency is small.	The efficiency score of carbon dioxide increases slightly, around 0.75. The AQI efficiency score drops from 1 in the previous two years to 0.72 in 2015 and 0.86 in 2016. AQI efficiency score is slightly better than CO_2_.	Should pay attention to carbon dioxide emissions and air pollutant emissions
Chongqing	Respiratory disease efficiency and mortality efficiency have risen, room for improvement is shrinking, and room for improvement in mortality efficiency is shrinking more.	The efficiency score of CO_2_ increases slightly, around 0.75. AQI efficiency score is 1 and the room for improvement is 0.	Priority carbon dioxide emission control
Fuzhou	The room for improvement is 0, indicating that these two indicators are more efficient than other cities.	The room for improvement of both indicators is 0.	
Guangzhou	The room for improvement is 0, indicating that these two indicators are more efficient than other cities.	The room for improvement of both indicators is 0.	
Guiyang	Respiratory disease efficiency and mortality efficiency do not change much, indicating that the improvement is not obvious.	The efficiency score of CO_2_ is rising but the room for improvement is large. In 2016, the efficiency score is only about 0.61. AQI efficiency score is better than CO_2_, only the efficiency score in 2015 is 0.65, and other years are 1	Emissions management of carbon dioxide should be prior to air pollutants.
Harbin	Fluctuation in the efficiency of respiratory disease efficiency and mortality efficiency, declines in 2014 and 2015, but returns to 2013 levels in 2016	CO_2_ efficiency score decreases, and it is 0.73 in 2016. The AQI efficiency score fluctuate greatly, but it has reached 1. in 2016. The situation is better than CO_2_.	Emissions management of carbon dioxide should be prior to air pollutants.
Haikou	The room for improvement is 0, indicating that these two indicators are more efficient than other cities.	The room for improvement of both indicators is 0.	
Hangzhou	Mortality efficiency improves significantly, and the room for improvement is reduced; respiratory disease improvement is not obvious, only a small increase.	The efficiency score of carbon dioxide fluctuates, but the efficiency score is 0.8 in 2016; AQI is 1 in the previous two years, but the decline in the last two years is larger, and the efficiency score in 2016 is 0.72.	Focus on carbon emissions and air pollution control. The efficiency of carbon emission improvement is not obvious, the room for improvement of air pollution is declining, and treatment should be strengthened
Hefei	Both efficiency scores fluctuate and rise to 1.	CO_2_ efficiency rises to 1 in 2016; AQI efficiency score increases, reaching 1 in 2016	Should pay attention to the management of carbon emissions and air pollution
Huhehot	Respiratory disease efficiency and mortality efficiency increase, but not much.	The CO_2_ efficiency is maintained at around 0.7, and there is room for improvement; AQI has some fluctuations, only 0.4 in 2015, but reached 1 in 2016.	Focus on carbon emissions and air pollution control. The efficiency improvement of carbon emissions is not obvious, and the efficiency of air pollution is not stable and should be paid attention to
Jinan	Both indicators have risen to 1 in 2016 and the room for improvement is 0.	The efficiency score of CO_2_ reaches 1 in the last year, but it is only about 0.56 in the first three years; AQI is only about 0.48 in 2015, and it has reached 1 in 2016.	Strengthen the treatment of CO2 emissions and air pollutant emissions
Kunming	The efficiency of respiratory diseases continues to decline, the efficiency score of mortality has fluctuated, and the mortality rate reached the highest in 2013.	The AQI efficiency score is significantly better than the efficiency score of carbon dioxide emissions.	Prioritize the control of CO2 emissions, but do not overlook the monitoring of air pollutant emissions.
Lanzhou	Respiratory disease and mortality efficiency have declined from the previous two years, but rose to above 0.9 in 2016.	The carbon dioxide emission efficiency score is lower than the AQI efficiency score for four years. There is room for improvement; AQI efficiency has dropped even more.	Focus on CO2 emissions and AQI
Lhasa	The room for improvement is 0, indicating that these two indicators are more efficient than other cities.	The room for improvement of both indicators is 0.	
Nanchang	The efficiency scores of the two indicators rises slightly.	The efficiency score of CO_2_ decreases slightly, and the AQI efficiency score decreases from 1 in the previous two years to around 0.62 in 2015 and rises to around 0.83 in 2016.	Focus on CO2 emissions and AQI
Nanjing	Respiratory disease efficiency increases slightly, mortality efficiency is greatly increased and the room for improvement is shrinking.	The CO_2_ efficiency score increases, and it is around 0.86 in 2016. The AQI efficiency score rises first and then falls, and it is only about 0.78 in 2016.	Focus on CO2 emissions and AQI
Nanning	The room for improvement in the efficiency of respiratory diseases is zero, and the room for improvement in mortality has been zero for the first three years, with a slight decline in the last year.	AQI efficiency improvement room is 0, but CO_2_ efficiency is 1 in three years, but falls to 0.88 in the last year.	Carbon dioxide emission control is prior and maintain control over air pollution emissions.
Shanghai	The room for improvement is 0, indicating that these two indicators are more efficient than other cities.	The room for improvement of both indicators is 0.	
Shenyang	Both indicators are slightly reduced, and the room for improvement is slightly expanded.	The emission efficiency of carbon dioxide has improved significantly, reaching 1 in 2016; AQI is only low in 2015, only 0.4, and the other three years are 1.	Focus on the control of carbon dioxide emissions and air pollution emissions
Shijiazhuang	Both indicators have declined, and the room for improvement has expanded slightly.	The CO_2_ efficiency score continues to decline, only about 0.39 in 2016. There is still room for improvement; AQI has also experiences a significant and sustained decline. By 2016, it is only about 0.42, and there is also room for improvement.	Strengthen the management of CO2 and air pollutant emissions, take comprehensive measures to reduce carbon emissions, and control pollutant emissions;
Taiyuan	Two efficiency scores rise slightly.	The carbon dioxide efficiency score has been below 0.2, and the efficiency of AQI has decreased. In 2016, it is only about 0.41.	Focus on the treatment of carbon dioxide emissions, but the emissions of air pollutants are also serious
Tianjin	Both efficiency drops.	The efficiency score of CO_2_ has not changed much, and it stays around 0.7, and it is only 0.67 in 2016; AQI declines, and it is only 0.66 in 2016.	Strengthen the management of carbon dioxide emissions and emissions of air pollution
Wuhan	Both indicators have fallen from 1 in 2013 and then increases slightly, but there is still room for improvement.	The efficiency score of CO_2_ decreases, and it is only about 0.74 in 2016, AQI efficiency score drops, and it is only around 0.5 in 2016.	Air pollution emission is prior to control CO2 emissions.
Urumqi	Respiratory efficiency and mortality efficiency have only room for improvement in 2015, and the room for improvement of other years is 0.	Both the CO_2_ efficiency score and the AQI efficiency score declines in 2015, 1 in other years, but AQI is only 0.33 in 2015.	Focus on carbon dioxide emissions and emissions of air pollutants
Xian	Both indicators have risen slightly	The change in carbon dioxide emissions is not obvious, and it remains at around 0.75; AQI is fluctuating, only about 0.65 in 2016.	Air pollutant emissions treatment first but should be integrated with carbon dioxide emissions.
Xining	Both indicators have risen, but the score for the last year of respiratory disease efficiency is comparable to 2013. The mortality efficiency has increased greatly.	The carbon dioxide efficiency score increases and reaches the highest level in 2016, but only about 0.56, AQI fluctuates and falls to around 0.48 in 2016.	Air pollutant emissions treatment first but should be integrated with carbon dioxide emissions.
Yinchuan	The efficiency of respiratory diseases increases slightly, while the mortality rate decreases slightly, and the mortality efficiency score is close to 1.	Carbon dioxide is falling, and the highest efficiency score is only about 0.37. By 2016, only 0.27. AQI efficiency score is slightly higher than CO_2_, but declines. By 2016, it is only about 0.44, and there is still room for improvement.	Air pollutant emissions treatment first but should be integrated with carbon dioxide emissions.
Zhengzhou	Respiratory efficiency decreases to 0.61 in 2016 and mortality efficiency score increases.	CO_2_ efficiency scores are better than AQI, but decline; AQI has fluctuated, and only 0.75 in 2016	Air pollutant emissions treatment first but should be integrated with carbon dioxide emissions.
